# Uniqueness of the Hadamard-type integral equations

**DOI:** 10.1186/s13662-020-03205-8

**Published:** 2021-01-09

**Authors:** Chenkuan Li

**Affiliations:** Department of Mathematics and Computer Science, Brandon University, Brandon, Manitoba R7A 6A9 Canada

**Keywords:** 45E10, 34A12, 26A33, Hadamard-type integral, Banach’s fixed point theorem, Babenko’s approach, Multivariate Mittag-Leffler function

## Abstract

The goal of this paper is to study the uniqueness of solutions of several Hadamard-type integral equations and a related coupled system in Banach spaces. The results obtained are new and based on Babenko’s approach and Banach’s contraction principle. We also present several examples for illustration of the main theorems.

## Introduction

The Hadamard-type fractional integral of order $\alpha > 0$ for a function *u* is defined in [1, 2] as

$$ \bigl(\mathcal{J}^{\alpha }_{ a + , \mu } u \bigr) (x) = \frac{1}{\Gamma (\alpha )} \int _{a}^{x} \biggl(\frac{t}{x} \biggr)^{\mu } \biggl(\log \frac{x}{t} \biggr)^{\alpha - 1} u(t) \frac{d t}{t}, $$

where $\log ( \cdot ) = \log _{e} (\cdot )$, $0 < a < x < b$, and $\mu \in R$. The corresponding derivative is given by

$$ \bigl(\mathcal{D}^{\alpha }_{a + , \mu } u \bigr) (x) = x^{-\mu } \delta ^{n} x^{\mu } \bigl(\mathcal{J}^{n - \alpha }_{ a + , \mu } u \bigr) (x), \quad \delta = x \frac{d}{d x}, $$

where $n = [\alpha ] + 1$, $[\alpha ]$ being the integral part of *α*. When $\mu = 0$, they take the forms

$$\begin{aligned}& \bigl(\mathcal{J}^{\alpha }_{ a + } u \bigr) (x) = \frac{1}{\Gamma (\alpha )} \int _{a}^{x} \biggl(\log \frac{x}{t} \biggr)^{\alpha - 1} u(t) \frac{d t}{t}, \\& \bigl(\mathcal{D}^{\alpha }_{ a + } u \bigr) (x) = \delta ^{n} \bigl(\mathcal{J}^{n - \alpha }_{ a + } u \bigr) (x), \end{aligned}$$

respectively. In particular, for $\alpha = 1$,

$$ (\mathcal{J}_{ a + , \mu } u) (x) = \bigl(\mathcal{J}^{1}_{ a + , \mu } u \bigr) (x) = \frac{1}{\Gamma (\alpha ) x^{\mu }} \int _{a}^{x} t^{ \mu - 1} u(t) \,d t, $$

which leads to definition of the space ${X}_{\mu }(a, b)$ of Lebesgue-measurable functions *u* on $[a, b]$ for which $x^{\mu - 1} u(x)$ is absolutely integrable [2]:

$$ X_{\mu }(a, b) = \biggl\{ u : [a, b] \rightarrow C: \lVert u \rVert _{X_{\mu }} = \int _{a}^{b} x^{\mu - 1} \bigl\vert u(x) \bigr\vert \,d x < \infty \biggr\} . $$

Clearly, for $a > 0$,

$$\begin{aligned}& \min_{x \in [a, b]} \bigl\{ x^{\mu - 1} \bigr\} \int _{a}^{b} \bigl\vert u(x) \bigr\vert \,d x \leq \int _{a}^{b} x^{\mu - 1} \bigl\vert u(x) \bigr\vert \,d x \leq \max_{x \in [a, b]} \bigl\{ x^{\mu - 1} \bigr\} \int _{a}^{b} \bigl\vert u(x) \bigr\vert \,d x, \quad \text{and} \\ & 0 < \min_{x \in [a, b]} \bigl\{ x^{\mu - 1} \bigr\} \leq \max _{x \in [a, b]} \bigl\{ x^{\mu - 1} \bigr\} \end{aligned}$$

for every $\mu \in R$. Hence $X_{\mu }(a, b)$ is a Banach space, since $L(a, b)$ with the norm

$$ \lVert u \rVert _{L} = \int _{a}^{b} \bigl\vert u(x) \bigr\vert \,d x $$

is complete and the norms $\lVert u \rVert _{X_{\mu }}$ and $\lVert u \rVert _{L} $ are equivalent.

We need the following lemmas shown by Kilbas [2].

### Lemma 1.1

*If* $\alpha > 0$, $\mu \in R$, *and* $0 < a < b < \infty $, *then the operator* $\mathcal{J}^{\alpha }_{ a + , \mu }$ *is bounded in* $X\mu (a, b)$, *and for* $u \in X\mu (a, b)$,

$$ \bigl\lVert \mathcal{J}^{\alpha }_{ a + , \mu } u \bigr\rVert _{X_{\mu }} \leq K \lVert u \rVert _{X_{\mu }}, $$

*where*

$$ K = \frac{1}{\Gamma (\alpha + 1)} \biggl[\log \biggl(\frac{b}{a} \biggr) \biggr]^{\alpha }. $$

### Lemma 1.2

*If* $\alpha >0$, $\beta > 0$, $\mu \in R$, *and* $u \in X_{\mu }(a, b)$, *then the semigroup property holds*:

$$ \mathcal{J}^{\alpha }_{ a + , \mu } \mathcal{J}^{\beta }_{ a + , \mu } u = \mathcal{J}^{\alpha + \beta }_{ a + , \mu }u. $$

There are a lot of studies on fractional differential and integral equations involving Riemann–Liouville or Caputo operators with boundary value problems or initial conditions [3–11]. Li and Sarwar [12] considered the existence of solutions for the following fractional-order initial value problems:

$$ \textstyle\begin{cases} ({}_{C} D_{0, t}^{\alpha }u) (t) = f(t, u(t)), \quad t \in (0, \infty ), \\ u(0) = u_{0}, \end{cases} $$

where $0 < \alpha < 1$, and ${}_{C}D_{0, t}^{\alpha }$ is the Caputo derivative.

Wu et al. [13] studied the existence and uniqueness of solutions by fixed point theory for the following fractional differential equation with nonlinearity depending on fractional derivatives of lower order on an infinite interval:

$$ \textstyle\begin{cases} (D_{0 +}^{\alpha }u) (t) + f(t, u(t), (D_{0 +}^{\alpha - 2} u) (t), (D_{0 +}^{\alpha - 1} u) (t)) = 0, \quad t \in (0, \infty ), \\ u(0) = u'(0) = 0, \quad\quad (D_{0 +}^{\alpha - 1} u) (\infty ) = \zeta , \end{cases} $$

where $2 < \alpha \leq 3$, $D_{0 +}^{\alpha }$, $D_{0 +}^{\alpha - 1}$, and $D_{0 +}^{\alpha - 2}$ are all Riemann–Liouville fractional derivatives.

Ahmad and Ntouyas [14] considered a coupled system of Hadamard-type fractional differential equations and integral boundary conditions

$$ \textstyle\begin{cases} \mathcal{D}^{\alpha }_{1 + } u (t) = w_{1}(t, u(t), v(t)), \quad 1 < t < e, 1 < \alpha \leq 2, \\ \mathcal{D}^{\beta }_{1 + } v (t) = w_{2}(t, u(t), v(t)), \quad 1 < t < e, 1 < \beta \leq 2, \\ u(1) = 0, \quad\quad u(e) = \mathcal{J}^{\gamma }_{ 1 + } u(\sigma _{1}) = \frac{1}{\Gamma (\gamma )} \int _{1}^{\sigma _{1}} (\log \frac{\sigma _{1}}{s} )^{\gamma - 1} u(s) \frac{d s}{s}, \\ v(1) = 0, \quad\quad v(e) = \mathcal{J}^{\gamma }_{ 1 + } v(\sigma _{2}) = \frac{1}{\Gamma (\gamma )} \int _{1}^{\sigma _{2}} (\log \frac{\sigma _{2}}{s} )^{\gamma - 1} v(s) \frac{d s}{s}, \end{cases} $$

where $\gamma > 0$, $1 < \sigma _{1} < e$, $1 < \sigma _{2} < e$, and $w_{1}, w_{2}: [1, e] \times R \times R \rightarrow R$ are continuous functions satisfying certain conditions. They showed the existence of solutions by Leray–Schauder’s alternative and the uniqueness by Banach’s fixed point theorem, based on the fact that for $1 < q \leq 2$ and $z \in C([1, e], R)$, the problem

$$ \textstyle\begin{cases} \mathcal{D}^{q}_{1 + } x (t) = z(t), \quad 1 < t < e, \\ x(1) = 0, \quad\quad x(e) = \mathcal{J}^{\gamma }_{ 1 + } x(\theta ), \end{cases} $$

has a unique solution

$$ x(t) = \mathcal{J}^{q}_{1 + } z (t) + \frac{(\log t)^{q - 1}}{Q} \bigl[ \mathcal{J}^{\gamma + q}_{ 1 + } z(\theta ) - \mathcal{J}^{q}_{ 1 + }z(e) \bigr], $$

where

$$ Q = \frac{1}{ 1 - \frac{1}{\Gamma (\gamma )} \int _{1}^{\theta } (\log \frac{\theta }{s} )^{\gamma - 1} (\log s)^{q - 1} \frac{d s}{s} }. $$

Let $g: [a, b] \times R \rightarrow R$ be a continuous function. In this paper, we study the following nonlinear Hadamard-type (*μ* is arbitrary in *R*) integral equation in the space $X_{\mu }(a, b)$:

1 $$ a_{n} \bigl(\mathcal{J}^{\alpha _{n}}_{ a + , \mu } u \bigr) (x) + \cdots + a_{1} \bigl(\mathcal{J}^{\alpha _{1}}_{ a + , \mu } u \bigr) (x) + u(x) = g \bigl(x, u(x) \bigr), $$

where $\alpha _{n} > \alpha _{n - 1} > \cdots > \alpha _{1} > 0$, and $a_{i}$, $i = 1, 2,\ldots,n$, are complex numbers, not all zero.

To the best of the author’s knowledge, equation (1) is new in the framework of Hadamard-type integral equations. First, by Babenko’s approach we will construct the solution as a convergent infinite series in $X_{\mu }(a, b)$ for the integral equation

2 $$ a_{n} \bigl(\mathcal{J}^{\alpha _{n}}_{ a + , \mu } u \bigr) (x) + \cdots + a_{1} \bigl(\mathcal{J}^{\alpha _{1}}_{ a + , \mu } u \bigr) (x) + u(x) = f(x), $$

where $f \in X_{\mu }(a, b)$. Then we will show that there exists a unique solution for equation (1) using Banach’s contraction principle. Furthermore, we present the solution for the Hadamard-type integral equation

3 $$ a_{n} \bigl(\mathcal{J}^{\alpha _{n}}_{ a + , \mu } u \bigr) (x) + \cdots + a_{1} \bigl(\mathcal{J}^{\alpha _{1}}_{ a + , \mu } u \bigr) (x) + \bigl(\mathcal{J}^{ \alpha _{0}}_{ a + , \mu } u \bigr) (x) = f(x) $$

by the Hadamard fractional derivative and show the uniqueness for the coupled system of integral equations

4 $$ \textstyle\begin{cases} a_{n} (\mathcal{J}^{\alpha _{n}}_{ a + , \mu } u)(x) + \cdots + a_{1} (\mathcal{J}^{\alpha _{1}}_{ a + , \mu } u)(x) + u(x) = g_{1}(x, u(x), v(x)), \\ b_{n} (\mathcal{J}^{\beta _{n}}_{ a + , \mu } v)(x) + \cdots + b_{1} (\mathcal{J}^{\beta _{1}}_{ a + , \mu } v)(x) + v(x) = g_{2}(x, u(x), v(x)), \end{cases} $$

where $\alpha _{n} > \alpha _{n - 1} > \cdots > \alpha _{1} > 0$, $\beta _{n} > \beta _{n - 1} > \cdots > \beta _{1} > 0$, and there exist at least one nonzero $a_{i}$ and one nonzero $b_{j}$ for some $1 \leq i, j \leq n$. We also present several examples for illustration of our results.

## Main results

We begin by showing the solution for equation (2) as a convergent series in the space $X_{\mu }(a, b)$ by Babenko’s approach [15], which is a powerful tool in solving differential and integral equations. The method itself is close to the Laplace transform method in the ordinary sense, but it can be used in more cases [16, 17], such as solving integral or fractional differential equations with distributions whose Laplace transforms do not exist in the classical sense. Clearly, it is always necessary to show the convergence of the series obtained as solutions. Podlubny [16] also provided interesting applications to solving certain partial differential equations for heat and mass transfer by Babenko’s method. Recently, Li and Plowman [18] and Li [19] studied the generalized Abel’s integral equations of the second kind with variable coefficients by Babenko’s technique.

### Theorem 2.1

*Let* $f \in X_{\mu }(a, b)$ *with* $0 < a < b < \infty $. *Then equation* (2) *has a unique solution in the space* $X_{\mu }(a, b)$,

5 $$ u(x) = \sum_{k = 0}^{\infty }(-1)^{k} \sum_{k_{1} + \cdots + k_{n} = k} \binom{k}{k_{1}, k_{2},\ldots, k_{n}} a_{n}^{k_{n}} \cdots a_{1}^{k_{1}} \bigl(\mathcal{J}^{k_{n} \alpha _{n} + \cdots + k_{1} \alpha _{1}}_{ a + , \mu } f \bigr) (x), $$

*where* $\alpha _{n} > \cdots > \alpha _{1} > 0$, *and* $a_{i}$, $i = 1, 2,\ldots, n$, *are complex numbers*, *not all zero*.

### Proof

Equation (2) can be written as

$$ \bigl(a_{n} \mathcal{J}^{\alpha _{n}}_{ a + , \mu } + \cdots + a_{1} \mathcal{J}^{\alpha _{1}}_{ a + , \mu } + 1 \bigr)u(x) = f(x). $$

By Babenko’s method we arrive at

$$\begin{aligned} u(x) = & \bigl(a_{n} \mathcal{J}^{\alpha _{n}}_{ a + , \mu } + \cdots + a_{1} \mathcal{J}^{\alpha _{1}}_{ a + , \mu } + 1 \bigr)^{-1} f(x) \\ = & \sum_{k = 0}^{\infty }(-1)^{k} \bigl(a_{n} \mathcal{J}^{\alpha _{n}}_{ a + , \mu } + \cdots + a_{1} \mathcal{J}^{\alpha _{1}}_{ a + , \mu } \bigr)^{k} f(x) \\ = & \sum_{k = 0}^{\infty }(-1)^{k} \sum_{k_{1} + \cdots + k_{n} = k} \binom{k}{k_{1}, k_{2},\ldots, k_{n}} \bigl(a_{n} \mathcal{J}^{\alpha _{n}}_{ a + , \mu } \bigr)^{k_{n}} \cdots \bigl(a_{1} \mathcal{J}^{\alpha _{1}}_{ a + , \mu } \bigr)^{k_{1}} f(x) \\ = & \sum_{k = 0}^{\infty }(-1)^{k} \sum_{k_{1} + \cdots + k_{n} = k} \binom{k}{k_{1}, k_{2},\ldots, k_{n}} a_{n}^{k_{n}} \mathcal{J}^{k_{n} \alpha _{n}}_{ a + , \mu } \cdots a_{1}^{k_{1}} \mathcal{J}^{k_{1} \alpha _{1}}_{ a + , \mu } f(x) \\ = & \sum_{k = 0}^{\infty }(-1)^{k} \sum_{k_{1} + \cdots + k_{n} = k} \binom{k}{k_{1}, k_{2},\ldots, k_{n}} a_{n}^{k_{n}} \cdots a_{1}^{k_{1}} \bigl(\mathcal{J}^{k_{n} \alpha _{n} + \cdots + k_{1} \alpha _{1}}_{ a + , \mu } f \bigr) (x) \end{aligned}$$

using Lemma 1.2 and the multinomial theorem. Note that

$$ \mathcal{J}^{0}_{ a + , \mu } f(x) = f(x). $$

It remains to show that series (5) converges in the space $X_{\mu }(a, b)$. By Lemma 1.1

$$ \bigl\lVert \mathcal{J}^{k_{n} \alpha _{n} + \cdots + k_{1} \alpha _{1}}_{ a + , \mu } f(x) \bigr\rVert _{X_{\mu }} \leq K \lVert f \rVert _{X_{\mu }}, $$

where

$$ K = \frac{1}{\Gamma (k_{n} \alpha _{n} + \cdots + k_{1} \alpha _{1} + 1)} \biggl(\log \frac{b}{a} \biggr)^{k_{n} \alpha _{n} + \cdots + k_{1} \alpha _{1}}. $$

Therefore

$$\begin{aligned}& \lVert u \rVert _{X\mu } \\& \quad \leq\sum_{k = 0}^{\infty }\sum _{k_{1} + \cdots + k_{n} = k} \binom{k}{k_{1}, k_{2},\ldots, k_{n}} \frac{ ( \vert a_{n} \vert (\log \frac{b}{a} )^{\alpha _{n}} )^{k_{n}} \cdots ( \vert a_{1} \vert (\log \frac{b}{a} )^{\alpha _{1}} )^{k_{1}} }{\Gamma (k_{n} \alpha _{n} + \cdots + k_{1} \alpha _{1} + 1)} \lVert f \rVert _{X_{\mu }} \\& \quad = E_{(\alpha _{1},\ldots, \alpha _{n}, 1)} \biggl( \vert a_{1} \vert \biggl(\log \frac{b}{a} \biggr)^{\alpha _{1}},\ldots, \vert a_{n} \vert \biggl(\log \frac{b}{a} \biggr)^{\alpha _{n}} \biggr) \lVert f \rVert _{X_{\mu }}, \end{aligned}$$

where

$$ E_{(\alpha _{1},\ldots, \alpha _{n}, 1)} \biggl( \vert a_{1} \vert \biggl( \log \frac{b}{a} \biggr)^{\alpha _{1}},\ldots, \vert a_{n} \vert \biggl(\log \frac{b}{a} \biggr)^{\alpha _{n}} \biggr) < \infty $$

is the value of the multivariate Mittag-Leffler function $E_{(\alpha _{1},\ldots, \alpha _{n}, 1)}(z_{1},\ldots, z_{n})$ given in [7] at

$$ z_{1} = \vert a_{1} \vert \biggl(\log \frac{b}{a} \biggr)^{\alpha _{1}},\quad\quad \ldots, \quad\quad z_{n} = \vert a_{n} \vert \biggl(\log \frac{b}{a} \biggr)^{\alpha _{n}}. $$

Thus $u \in X_{\mu }(a, b)$, and the series on the right-hand of equation (5) is convergent.

To verify that the series is a solution, we substitute it into the left-hand side of equation (2):

$$\begin{aligned}& a_{n} \sum_{k = 0}^{\infty }(-1)^{k} \sum_{k_{1} + \cdots + k_{n} = k} \binom{k}{k_{1}, k_{2},\ldots, k_{n}} a_{n}^{k_{n}} \cdots a_{1}^{k_{1}} \bigl(\mathcal{J}^{(k_{n} + 1) \alpha _{n} + \cdots + k_{1} \alpha _{1}}_{ a + , \mu } f \bigr) (x) + \cdots \\& \quad\quad{}+ a_{1} \sum_{k = 0}^{\infty }(-1)^{k} \sum_{k_{1} + \cdots + k_{n} = k} \binom{k}{k_{1}, k_{2},\ldots, k_{n}} a_{n}^{k_{n}} \cdots a_{1}^{k_{1}} \bigl(\mathcal{J}^{k_{n} \alpha _{n} + \cdots + (k_{1} + 1) \alpha _{1}}_{ a + , \mu } f \bigr) (x) + \cdots \\& \quad \quad{}+ \sum_{k = 0}^{\infty }(-1)^{k} \sum_{k_{1} + \cdots + k_{n} = k} \binom{k}{k_{1}, k_{2},\ldots, k_{n}} a_{n}^{k_{n}} \cdots a_{1}^{k_{1}} \bigl(\mathcal{J}^{k_{n} \alpha _{n} + \cdots + k_{1} \alpha _{1}}_{ a + , \mu } f \bigr) (x) \\& \quad = a_{n} \bigl(\mathcal{J}^{\alpha _{n}}_{a+, \mu }f \bigr) (x) + a_{n} \sum_{k = 1}^{\infty }(-1)^{k} \sum_{k_{1} + \cdots + k_{n} = k} \binom{k}{k_{1}, k_{2},\ldots, k_{n}}a_{n}^{k_{n}} \cdots a_{1}^{k_{1}} \\& \quad \quad {}\cdot \bigl(\mathcal{J}^{(k_{n} + 1) \alpha _{n} + \cdots + k_{1} \alpha _{1}}_{ a + , \mu } f \bigr) (x) + \cdots + a_{1} \bigl(\mathcal{J}^{\alpha _{1}}_{a+, \mu }f \bigr) (x) \\& \quad \quad {}+a_{1} \sum_{k = 1}^{\infty }(-1)^{k} \sum_{k_{1} + \cdots + k_{n} = k} \binom{k}{k_{1}, k_{2},\ldots, k_{n}} a_{n}^{k_{n}} \cdots a_{1}^{k_{1}} \bigl(\mathcal{J}^{k_{n} \alpha _{n} + \cdots + (k_{1} + 1) \alpha _{1}}_{ a + , \mu } f \bigr) (x) \\& \quad \quad {}+f(x) - a_{n} \bigl(\mathcal{J}^{\alpha _{n}}_{a+, \mu }f \bigr) (x) - \cdots - a_{1} \bigl(\mathcal{J}^{\alpha _{1}}_{a+, \mu }f \bigr) (x) \\& \quad \quad {} + \sum_{k = 2}^{\infty }(-1)^{k} \sum_{k_{1} + \cdots + k_{n} = k} \binom{k}{k_{1}, k_{2},\ldots, k_{n}} a_{n}^{k_{n}} \cdots a_{1}^{k_{1}} \bigl(\mathcal{J}^{k_{n} \alpha _{n} + \cdots + k_{1} \alpha _{1}}_{ a + , \mu } f \bigr) (x) \\& \quad = a_{n} \sum_{k = 1}^{\infty }(-1)^{k} \sum_{k_{1} + \cdots + k_{n} = k} \binom{k}{k_{1}, k_{2},\ldots, k_{n}} a_{n}^{k_{n}} \cdots a_{1}^{k_{1}} \bigl(\mathcal{J}^{(k_{n} + 1) \alpha _{n} + \cdots + k_{1} \alpha _{1}}_{ a + , \mu } f \bigr) (x)+ \cdots \\& \quad \quad {}+a_{1} \sum_{k = 1}^{\infty }(-1)^{k} \sum_{k_{1} + \cdots + k_{n} = k} \binom{k}{k_{1}, k_{2},\ldots, k_{n}} a_{n}^{k_{n}} \cdots a_{1}^{k_{1}} \bigl(\mathcal{J}^{k_{n} \alpha _{n} + \cdots + (k_{1} + 1) \alpha _{1}}_{ a + , \mu } f \bigr) (x) \\& \quad \quad{}+ f(x) + \sum_{k = 2}^{\infty }(-1)^{k} \sum_{k_{1} + \cdots + k_{n} = k} \binom{k}{k_{1}, k_{2},\ldots, k_{n}} a_{n}^{k_{n}} \cdots a_{1}^{k_{1}} \bigl(\mathcal{J}^{k_{n} \alpha _{n} + \cdots + k_{1} \alpha _{1}}_{ a + , \mu } f \bigr) (x) = f(x) \end{aligned}$$

as

$$\begin{aligned}& a_{n} \sum_{k = 1}^{\infty }(-1)^{k} \sum_{k_{1} + \cdots + k_{n} = k} \binom{k}{k_{1}, k_{2},\ldots, k_{n}} a_{n}^{k_{n}} \cdots a_{1}^{k_{1}} \bigl(\mathcal{J}^{(k_{n} + 1) \alpha _{n} + \cdots + k_{1} \alpha _{1}}_{ a + , \mu } f \bigr) (x) + \cdots \\& \quad{}+ a_{1} \sum_{k = 1}^{\infty }(-1)^{k} \sum_{k_{1} + \cdots + k_{n} = k} \binom{k}{k_{1}, k_{2},\ldots, k_{n}} a_{n}^{k_{n}} \cdots a_{1}^{k_{1}} \bigl(\mathcal{J}^{k_{n} \alpha _{n} + \cdots + (k_{1} + 1) \alpha _{1}}_{ a + , \mu } f \bigr) (x) \\& \quad{}+ \sum_{k = 2}^{\infty }(-1)^{k} \sum_{k_{1} + \cdots + k_{n} = k} \binom{k}{k_{1}, k_{2},\ldots, k_{n}} a_{n}^{k_{n}} \cdots a_{1}^{k_{1}} \bigl(\mathcal{J}^{k_{n} \alpha _{n} + \cdots + k_{1} \alpha _{1}}_{ a + , \mu } f \bigr) (x) = 0 \end{aligned}$$

by cancelation. Note that all series are absolutely convergent and the term rearrangements are feasible for cancelation.

Clearly, the uniqueness immediately follows from the fact that the integral equation

$$ a_{n} \bigl(\mathcal{J}^{\alpha _{n}}_{ a + , \mu } u \bigr) (x) + \cdots + a_{1} \bigl(\mathcal{J}^{\alpha _{1}}_{ a + , \mu } u \bigr) (x) + u(x) = 0 $$

only has zero solution by Babenko’s method. This completes the proof of Theorem 2.1. □

Let $\nu > 0$ and $x \geq 0$. The incomplete gamma function is defined by

$$ \gamma (\nu , x) = \int _{0}^{x} t^{\nu - 1} e^{-t} \,dt. $$

From the recurrence relation [20]

$$ \gamma ( \nu + 1, x) = \nu \gamma (\nu , x) - x^{\nu }e ^{-x} $$

we get

6 $$ \gamma (\nu , x) = x^{\nu }\Gamma (\nu ) e^{-x} \sum_{j = 0}^{\infty }\frac{x^{j}}{\Gamma (\nu + j + 1)}. $$

### Example 1

Let $0< a < x < b$. Then the Hadamard-type integral equation

$$ \bigl(\mathcal{J}^{\frac{1}{2}}_{ a + , -1} u \bigr) (x) + u(x) = x^{2} $$

has the solution

$$ u(x) = a x \sum_{k = 0}^{\infty }\sum _{j = 0}^{\infty }\frac{ (-1)^{k} (\log \frac{x}{a} )^{j + \frac{1}{2} k}}{ \Gamma (\frac{1}{2} k + j +1 )}. $$

Indeed, it follows from Lemma 2.4 in [2] that

$$ \bigl(\mathcal{J}^{\alpha }_{ a + , \mu } t^{w} \bigr) (x) = \frac{\gamma (\alpha , (\mu + w) \log (x/a))}{\Gamma (\alpha )} (\mu + w)^{-\alpha } x^{w}, $$

where $\mu + w > 0$.

By Theorem 2.1

$$\begin{aligned} u(x) = & \sum_{k = 0}^{\infty }(-1)^{k} \bigl(\mathcal{J}^{\frac{1}{2} k}_{ a + , - 1} t^{2} \bigr) (x) = x^{2} \sum_{k = 0}^{\infty }(-1)^{k} \frac{\gamma (k/2, \log (x/a))}{\Gamma (k/2)}. \end{aligned}$$

Applying equation (6), we have

$$ \gamma \bigl(k/2, \log (x/a) \bigr) = (\log x/a )^{k/2} \Gamma (k/2) \frac{a}{x} \sum_{j = 0}^{\infty } \frac{ (\log x/a )^{j}}{\Gamma (\frac{1}{2} k + j + 1)}. $$

Thus

$$ u(x) = a x \sum_{k = 0}^{\infty }\sum _{j = 0}^{\infty }\frac{ (-1)^{k} (\log \frac{x}{a} )^{j + \frac{1}{2} k}}{ \Gamma (\frac{1}{2} k + j +1 )} $$

is a solution in the space $X_{\mu }(a, b)$.

The following theorem shows the uniqueness of solution of equation (1).

### Theorem 2.2

*Let* $g: [a, b] \times R \rightarrow R$ *be a continuous function and suppose that there exists a constant* $C > 0$ *such that for all* $x \in [a, b]$,

$$ \bigl\vert g(x, y_{1}) - g(x, y_{2}) \bigr\vert \leq C \vert y_{1} - y_{2} \vert , \quad y_{1}, y_{2} \in R. $$

*Furthermore*, *suppose that*

$$ C E_{(\alpha _{1},\ldots, \alpha _{n}, 1)} \biggl( \vert a_{1} \vert \biggl( \log \frac{b}{a} \biggr)^{\alpha _{1}},\ldots, \vert a_{n} \vert \biggl(\log \frac{b}{a} \biggr)^{\alpha _{n}} \biggr) < 1. $$

*Then equation* (1) *has a unique solution in the space* $X_{\mu }(a, b)$ *for every* $\mu \in R$.

### Proof

Let $u \in X_{\mu }(a, b)$. Then $g (x, u(x)) \in X_{\mu }(a, b)$ since

$$ \bigl\vert g \bigl(x, u(x) \bigr) \bigr\vert \leq \bigl\vert g \bigl(x, u(x) \bigr) - g(x, 0) \bigr\vert + \bigl\vert g(x, 0) \bigr\vert \leq C \bigl\vert u(x) \bigr\vert + \bigl\vert g(x, 0) \bigr\vert \in X_{\mu }(a, b) $$

by noting that $g(x, 0)$ is a continuous function on $[a, b]$. Define the mapping *T* on $X_{\mu }(a, b)$ by

$$ T(u) (x) = \sum_{k = 0}^{\infty }(-1)^{k} \sum_{k_{1} + \cdots + k_{n} = k} \binom{k}{k_{1}, k_{2},\ldots, k_{n}} a_{n}^{k_{n}} \cdots a_{1}^{k_{1}} \bigl(\mathcal{J}^{k_{n} \alpha _{n} + \cdots + k_{1} \alpha _{1}}_{ a + , \mu } g \bigl(t, u(t) \bigr) \bigr) (x). $$

In particular, for $k = 0$,

$$ \mathcal{J}^{k_{n} \alpha _{n} + \cdots + k_{1} \alpha _{1}}_{ a + , \mu } g \bigl(t, u(t) \bigr) (x) = g \bigl(x, u(x) \bigr). $$

From the proof of Theorem 2.1 we have

$$\begin{aligned} \bigl\lVert T(u) \bigr\rVert _{X\mu } \leq E_{(\alpha _{1},\ldots, \alpha _{n}, 1)} \biggl( \vert a_{1} \vert \biggl(\log \frac{b}{a} \biggr)^{ \alpha _{1}},\ldots, \vert a_{n} \vert \biggl(\log \frac{b}{a} \biggr)^{ \alpha _{n}} \biggr) \bigl\lVert g \bigl(x, u(x) \bigr) \bigr\rVert _{X_{\mu }}. \end{aligned}$$

Clearly,

$$ \bigl\lVert g \bigl(x, u(x) \bigr) \bigr\rVert _{X_{\mu }} \leq C \lVert u \rVert _{X_{\mu }} + \max_{x \in [a, b]} \bigl\{ x^{\mu - 1} \bigl\vert g (x, 0) \bigr\vert \bigr\} (b - a) < \infty . $$

Hence *T* is a mapping from $X_{\mu }(a, b)$ to $X_{\mu }(a, b)$. It remains to prove that *T* is contractive. We have

$$\begin{aligned} \bigl\lVert T(u) - T(v) \bigr\rVert _{X_{\mu }} \leq &\sum _{k = 0}^{\infty }\sum_{k_{1} + \cdots + k_{n} = k} \binom{k}{k_{1}, k_{2},\ldots, k_{n}} \\ & {}\cdot \vert a_{n} \vert ^{k_{n}} \cdots \vert a_{1} \vert ^{k_{1}} \bigl\lVert \mathcal{J}^{k_{n} \alpha _{n} + \cdots + k_{1} \alpha _{1}}_{ a + , \mu } \bigl(g \bigl(t, u(t) \bigr) - g \bigl(t, v(t) \bigr) \bigr) (x) \bigr\rVert _{X_{m}u} \\ \leq& \sum_{k = 0}^{\infty }\sum _{k_{1} + \cdots + k_{n} = k} \binom{k}{k_{1}, k_{2},\ldots, k_{n}} \frac{ ( \vert a_{n} \vert (\log \frac{b}{a} )^{\alpha _{n}} )^{k_{n}} \cdots ( \vert a_{1} \vert (\log \frac{b}{a} )^{\alpha _{1}} )^{k_{1}} }{\Gamma (k_{n} \alpha _{n} + \cdots + k_{1} \alpha _{1} + 1)} \\ & {}\cdot \bigl\lVert g \bigl(t, u(t) \bigr) - g \bigl(t, v(t) \bigr) \bigr\rVert _{X_{\mu }}. \end{aligned}$$

Since

$$ \bigl\lVert g \bigl(t, u(t) \bigr) - g \bigl(t, v(t) \bigr) \bigr\rVert _{X_{\mu }} \leq C \lVert u - v \rVert _{X_{\mu }}, $$

we derive

$$ \bigl\lVert T(u) - T(v) \bigr\rVert _{X_{\mu }} \leq C E_{(\alpha _{1},\ldots, \alpha _{n}, 1)} \biggl( \vert a_{1} \vert \biggl(\log \frac{b}{a} \biggr)^{\alpha _{1}},\ldots, \vert a_{n} \vert \biggl(\log \frac{b}{a} \biggr)^{\alpha _{n}} \biggr) \lVert u - v \rVert _{X_{\mu }}. $$

Therefore *T* is contractive. This completes the proof of Theorem 2.2. □

### Example 2

Let $a = 1$, $b = e$, and $\mu \in R$. Then for every $\mu \in R$, there is a unique solution for the following Hadamard-type integral equation:

7 $$ \bigl(\mathcal{J}^{1.5}_{ 1 + , \mu } u \bigr) (x) + ( \mathcal{J}_{ 1 + , \mu } u) (x) + u(x) = \frac{x^{2}}{9(1 + x^{2})} \sin u(x) + \cos ( \sin x) + 1. $$

Clearly, the function

$$ g(x , y) = \frac{x^{2}}{9(1 + x^{2})} \sin y + \cos (\sin x) + 1 $$

is a continuous function from $[1, e] \times R$ to *R* and satisfies

$$ \bigl\vert g(x, y_{1}) - g(x, y_{2}) \bigr\vert \leq \frac{x^{2}}{9(1 + x^{2})} \vert \sin y_{1} - \sin y_{2} \vert \leq \frac{x^{2}}{9(1 + x^{2})} \vert y_{1} - y_{2} \vert \leq \frac{1}{9} \vert y_{1} - y_{2} \vert . $$

Obviously, $a_{2} = a_{1} = 1$, and $\log b/a = 1$. By Theorem 2.2 we need to calculate the value

$$\begin{aligned} \sum_{k = 0}^{\infty }\sum _{k_{1} + k_{2} = k} \binom{k}{k_{1}, k_{2}} \frac{1}{\Gamma (1.5 k_{2} + k_{1} + 1)} =& \sum _{k = 0}^{\infty }\sum_{j = 0}^{k} \binom{k}{j} \frac{1}{\Gamma (k + 1 + 0.5 j)} \\ = &1 + \sum_{k = 1}^{\infty }\sum _{j = 0}^{k} \binom{k}{j} \frac{1}{\Gamma (k + 1 + 0.5 j)}. \end{aligned}$$

For $k \geq 1$ and $j \geq 0$, we have

$$ \frac{1}{\Gamma (k + 1 + 0.5 j)} \leq \frac{1}{\Gamma (k + 1) } = \frac{1}{k!} \quad \text{and} \quad \sum_{j = 0}^{k} \binom{k}{j} = 2^{k}. $$

Therefore

$$\begin{aligned}& \sum_{k = 0}^{\infty }\sum _{j = 0}^{k} \binom{k}{j} \frac{1}{\Gamma (1.5 k_{2} + k_{1} + 1)} \\& \quad \leq 1 + \sum_{k = 1}^{\infty }\frac{2^{k}}{k!} \\& \quad = 1 + 2 + \frac{2 \cdot 2}{1 \cdot 2} + \frac{2 \cdot 2 \cdot 2}{1 \cdot 2 \cdot 3} + \frac{2 \cdot 2 \cdot 2 \cdot 2}{1 \cdot 2 \cdot 3 \cdot 4} + \frac{2 \cdot 2 \cdot 2 \cdot 2 \cdot 2}{1 \cdot 2 \cdot 3 \cdot 4 \cdot 5} + \cdots \\& \quad \leq 1 + 2 + 2 + \biggl( \frac{1}{3} + \biggl(\frac{2}{3} \biggr)^{0} \biggr) + \biggl(\frac{2}{3} \biggr)^{1} + \biggl(\frac{2}{3} \biggr)^{2} + \cdots \\& \quad = \frac{16}{3} + \frac{1}{1 - \frac{2}{3}} = \frac{25}{3}. \end{aligned}$$

Then

$$ C \sum_{k = 0}^{\infty }\sum _{k_{1} + k_{2} = k} \binom{k}{k_{1}, k_{2}} \frac{1}{\Gamma (1.5 k_{2} + k_{1} + 1)} < \frac{25}{3} \cdot \frac{1}{9} < 1. $$

By Theorem 2.2 equation (7) has a unique solution.

### Remark 1

There are algorithms for computation of the Mittag-Leffler function [21]

$$ E_{\alpha , \beta }(z) = \sum_{k = 0}^{\infty } \frac{z^{k}}{\Gamma (\alpha k + \beta )}, \quad \alpha > 0, \beta \in R, z \in C, $$

and its derivative. In particular,

$$ E_{\alpha , \beta }(z) = - \frac{\sin (\pi \alpha )}{\pi \alpha } \int _{0}^{\infty }\frac{e^{-r^{1/\alpha }}}{r^{2} - 2 rz \cos (\pi \alpha ) + z^{2}} \,d r - \frac{1}{z}, \beta = 1 + \alpha , $$

where $0 < \alpha \leq 1$, $\beta \in R$, $\vert \arg z \vert > \pi \alpha $, $z \neq 0$.

The Mittag-Leffler function is widely used in studying fractional differential equations and fractional calculus. Li [22] studied three classes of fractional oscillators and obtained the solutions of the first class in terms of the Mittag-Leffler function.

Define the product space $X_{\mu }(a, b) \times X_{\mu }(a, b)$ with the norm

$$ \bigl\lVert (u, v) \bigr\rVert = \lVert u \rVert _{X_{\mu }} + \lVert v \rVert _{X_{\mu }}. $$

Clearly, $X_{\mu }(a, b) \times X_{\mu }(a, b)$ is a Banach space.

Now we can extend Theorem 2.2 to the coupled system of the Hadamard-type integral equations given by (4).

### Theorem 2.3

*Let* $g_{1}, g_{2}: [a, b] \times R \times R \rightarrow R$ *be continuous functions and suppose that there exist nonnegative constants* $C_{i}$, $i = 1, 2, 3, 4$, *such that for all* $x \in [a, b]$ *and* $u_{i}, v_{i} \in R$, $i = 1, 2$,

$$\begin{aligned}& \bigl\vert g_{1}(x, u_{1}, v_{1}) - g_{1}(x, u_{2}, v_{2}) \bigr\vert \leq C_{1} \vert u_{1} - u_{2} \vert + C_{2} \vert v_{1} - v_{2} \vert , \\& \bigl\vert g_{2}(x, u_{1}, v_{1}) - g_{2}(x, u_{2}, v_{2}) \bigr\vert \leq C_{3} \vert u_{1} - u_{2} \vert + C_{4} \vert v_{1} - v_{2} \vert . \end{aligned}$$

*Furthermore*, *suppose that*

$$\begin{aligned} q = & \max \{C_{1}, C_{2}\} E_{(\alpha _{1},\ldots, \alpha _{n}, 1)} \biggl( \vert a_{1} \vert \biggl(\log \frac{b}{a} \biggr)^{\alpha _{1}},\ldots, \vert a_{n} \vert \biggl(\log \frac{b}{a} \biggr)^{\alpha _{n}} \biggr) \\ & {} + \max \{C_{3}, C_{4}\} E_{(\beta _{1},\ldots, \beta _{n}, 1)} \biggl( \vert b_{1} \vert \biggl(\log \frac{b}{a} \biggr)^{\beta _{1}},\ldots, \vert b_{n} \vert \biggl(\log \frac{b}{a} \biggr)^{\beta _{n}} \biggr) < 1. \end{aligned}$$

*Then system* (4) *has a unique solution in the product space* $X_{\mu }(a, b) \times X_{\mu }(a, b)$ *for every* $\mu \in R$.

### Proof

Let $u, v \in X_{\mu }(a, b)$. Then $g_{1}(x, u(x), v(x)), g_{2}(x, u(x), v(x)) \in X_{\mu }(a, b)$ since

$$\begin{aligned} \bigl\vert g_{1} \bigl(x, u(x), v(x) \bigr) \bigr\vert \leq& \bigl\vert g_{1} \bigl(x, u(x), v(x) \bigr) - g_{1}(x, 0, 0) \bigr\vert + \bigl\vert g_{1}(x, 0, 0) \bigr\vert \\ \leq &C_{1} \bigl\vert u(x) \bigr\vert + C_{2} \bigl\vert v(x) \bigr\vert + \bigl\vert g_{1}(x, 0, 0) \bigr\vert \in X_{\mu }(a, b) \end{aligned}$$

by noting that $g_{1}(x, 0, 0)$ is a continuous function on $[a, b]$. Furthermore,

$$ \bigl\lVert g_{1} \bigl(x, u(x), v(x) \bigr) \bigr\rVert _{X_{\mu }} \leq C_{1} \lVert u \rVert _{X_{\mu }} + C_{2} \lVert v \rVert _{X_{\mu }} + \max_{x \in [a, b]} \bigl\{ x^{\mu - 1} \bigl\vert g_{1}(x, 0, 0) \bigr\vert \bigr\} (b - a) < \infty $$

for every $\mu \in R$.

Define the mapping *T* on $X_{\mu }(a, b) \times X_{\mu }(a, b)$ by

$$ T(u, v) = \bigl(T_{1}(u, v), T_{2}(u, v) \bigr), $$

where

$$\begin{aligned} T_{1}(u, v) (x) = & \sum_{k = 0}^{\infty }(-1)^{k} \sum_{k_{1} + \cdots + k_{n} = k} \binom{k}{k_{1}, k_{2},\ldots, k_{n}}a_{n}^{k_{n}} \cdots a_{1}^{k_{1}} \\ & {}\cdot \mathcal{J}^{k_{n} \alpha _{n} + \cdots + k_{1} \alpha _{1}}_{ a + , \mu } g_{1} \bigl(t, u(t), v(t) \bigr) (x), \end{aligned}$$

and

$$\begin{aligned} T_{2}(u, v) (x) = & \sum_{k = 0}^{\infty }(-1)^{k} \sum_{k_{1} + \cdots + k_{n} = k} \binom{k}{k_{1}, k_{2},\ldots, k_{n}}b_{n}^{k_{n}} \cdots b_{1}^{k_{1}} \\ & {} \cdot\mathcal{J}^{k_{n} \beta _{n} + \cdots + k_{1} \beta _{1}}_{ a + , \mu } g_{2} \bigl(t, u(t), v(t) \bigr) (x). \end{aligned}$$

Clearly, from the proof of Theorem 2.2 we have

$$\begin{aligned} \bigl\lVert T_{1}(u, v) \bigr\rVert _{X\mu } \leq & E_{(\alpha _{1},\ldots, \alpha _{n}, 1)} \biggl( \vert a_{1} \vert \biggl(\log \frac{b}{a} \biggr)^{\alpha _{1}},\ldots, \vert a_{n} \vert \biggl(\log \frac{b}{a} \biggr)^{\alpha _{n}} \biggr) \\ & {}\cdot \Bigl(C_{1} \lVert u \rVert _{X_{\mu }} + C_{2} \lVert v \rVert _{X_{\mu }} + \max_{x \in [a, b]} \bigl\{ x^{\mu - 1} \bigl\vert g_{1} (x, 0, 0) \bigr\vert \bigr\} (b - a) \Bigr)< \infty \end{aligned}$$

and

$$\begin{aligned} \bigl\lVert T_{2}(u, v) \bigr\rVert _{X\mu } \leq & E_{(\beta _{1},\ldots, \beta _{n}, 1)} \biggl( \vert b_{1} \vert \biggl(\log \frac{b}{a} \biggr)^{\beta _{1}},\ldots, \vert b_{n} \vert \biggl(\log \frac{b}{a} \biggr)^{\beta _{n}} \biggr) \\ &{}\cdot \Bigl(C_{3} \lVert u \rVert _{X_{\mu }} + C_{4} \lVert v \rVert _{X_{\mu }} + \max_{x \in [a, b]} \bigl\{ x^{\mu - 1} \bigl\vert g_{2} (x, 0, 0) \bigr\vert \bigr\} (b - a) \Bigr)< \infty . \end{aligned}$$

Hence

$$ \bigl\lVert T(u, v) \bigr\rVert = \bigl\lVert T_{1}(u, v) \bigr\rVert _{X\mu } + \bigl\lVert T_{2}(u, v) \bigr\rVert _{X\mu } < \infty , $$

which implies that *T* maps the Banach space $X_{\mu }(a, b) \times X_{\mu }(a, b)$ into itself. It remains to show that *T* is contractive. Indeed,

$$\begin{aligned} \bigl\lVert T_{1}(u_{1}, v_{1}) - T_{1}(u_{2}, v_{2}) \bigr\rVert _{X_{\mu }} \leq & E_{(\alpha _{1},\ldots, \alpha _{n}, 1)} \biggl( \vert a_{1} \vert \biggl(\log \frac{b}{a} \biggr)^{\alpha _{1}},\ldots, \vert a_{n} \vert \biggl(\log \frac{b}{a} \biggr)^{\alpha _{n}} \biggr) \\ & {}\cdot\max \{C_{1}, C_{2}\} \bigl( \lVert u_{1} - u_{2} \rVert _{X_{\mu }} + \lVert v_{1} - v_{2} \rVert _{X_{\mu }} \bigr), \end{aligned}$$

and

$$\begin{aligned} \bigl\lVert T_{2}(u_{1}, v_{1}) - T_{2}(u_{2}, v_{2}) \bigr\rVert _{X_{\mu }} \leq & E_{(\beta _{1},\ldots, \beta _{n}, 1)} \biggl( \vert b_{1} \vert \biggl(\log \frac{b}{a} \biggr)^{\beta _{1}},\ldots, \vert b_{n} \vert \biggl( \log \frac{b}{a} \biggr)^{\beta _{n}} \biggr) \\ & {}\cdot\max \{C_{3}, C_{4}\} \bigl( \lVert u_{1} - u_{2} \rVert _{X_{\mu }} + \lVert v_{1} - v_{2} \rVert _{X_{\mu }} \bigr). \end{aligned}$$

Thus

$$\begin{aligned} \bigl\lVert T(u_{1}, v_{1}) - T(u_{2}, v_{2}) \bigr\rVert = & \bigl\lVert T_{1}(u_{1}, v_{1}) - T_{1}(u_{2}, v_{2}) \bigr\rVert _{X \mu } + \bigl\lVert T_{2}(u_{1}, v_{1}) - T_{2}(u_{2}, v_{2}) \bigr\rVert _{X\mu } \\ \leq &q \bigl( \lVert u_{1} - u_{2} \rVert _{X_{\mu }} + \lVert v_{1} - v_{2} \rVert _{X_{\mu }} \bigr), \end{aligned}$$

where $q < 1$ by assumption. By Banach’s contractive principle system (4) has a unique solution in the space $X_{\mu }(a, b) \times X_{\mu }(a, b)$. This completes the proof of Theorem 2.3. □

Let $\operatorname{AC}[a, b]$ be the set of absolutely continuous functions on $[a, b]$, which coincides with the space of primitives of Lebesgue-measurable functions [3]:

$$ h \in \operatorname{AC}[a, b] \quad \text{if and only if} \quad h(x) = h(a) + \int _{a}^{x} \psi (t) \,d t, \quad \psi \in L[a, b]. $$

Clearly, if $f \in \operatorname{AC}[a, b]$ with $0 < a < b < \infty $, then $x^{\mu } f(x) \in \operatorname{AC}[a, b]$ since $x^{\mu }\in \operatorname{AC}[a, b]$.

The following results are from Lemma 2.3 and Theorem 5.5(a) in [2].

(i) If $\alpha > \beta > 0$ and $\mu \in R$, then for $u \in X_{\mu }(a, b)$,
  $$ \mathcal{D}^{\beta }_{a + , \mu } \mathcal{J}^{\alpha }_{a + , \mu }u = \mathcal{J}^{\alpha - \beta }_{a + , \mu }u. $$
(ii) If $\alpha > 0$ and $u \in X_{\mu }(a, b)$, then
  $$ \mathcal{D}^{\alpha }_{a + , \mu } \mathcal{J}^{\alpha }_{a + , \mu }u = u. $$

### Theorem 2.4

*Let* $\alpha _{n} > \cdots > \alpha _{1} > \alpha _{0}$ *with* $0 < \alpha _{0} < 1$, *and let* $f \in \operatorname{AC}[a, b]$. *In addition*, *let* $a_{i}$, $i = 1, 2,\ldots, n$, *be complex numbers*, *not all zero*. *Then equation* (3) *has a unique solution in the space* $X_{\mu }(a, b)$,

$$\begin{aligned} u(x) = & a^{\mu }f(a) x^{- \mu } \biggl(\log \frac{x}{a} \biggr)^{ - \alpha _{0}} \sum_{k = 0}^{\infty }(-1)^{k} \sum_{k_{1} + \cdots + k_{n} = k} \binom{k}{k_{1}, k_{2},\ldots, k_{n}} a_{n}^{k_{n}} \cdots a_{1}^{k_{1}} \\ & {}\cdot\frac{ (\log \frac{x}{a} )^{ k_{n}(\alpha _{n} - \alpha _{0}) + \cdots + k_{1} (\alpha _{1} - \alpha _{0})}}{\Gamma (k_{n}(\alpha _{n} - \alpha _{0}) + \cdots + k_{1} (\alpha _{1} - \alpha _{0}) + 1 - \alpha _{0})} \\ & {} + \mu \sum_{k = 0}^{\infty }(-1)^{k} \sum_{k_{1} + \cdots + k_{n} = k} \binom{k}{k_{1}, k_{2},\ldots, k_{n}} a_{n}^{k_{n}} \cdots a_{1}^{k_{1}} \\ &{}\cdot \bigl(\mathcal{J}^{k_{n} (\alpha _{n} - \alpha _{0}) + \cdots + k_{1} ( \alpha _{1} - \alpha _{0}) + 1 - \alpha _{0} }_{ a + , \mu } f \bigr) (x) \\ & {} + \sum_{k = 0}^{\infty }(-1)^{k} \sum_{k_{1} + \cdots + k_{n} = k} \binom{k}{k_{1}, k_{2},\ldots, k_{n}} a_{n}^{k_{n}} \cdots a_{1}^{k_{1}} \\ & {}\cdot \bigl(\mathcal{J}^{k_{n} (\alpha _{n} - \alpha _{0}) + \cdots + k_{1} ( \alpha _{1} - \alpha _{0}) + 1 - \alpha _{0} }_{ a + , \mu } t f'(t) \bigr) (x). \end{aligned}$$

### Proof

It follows from Theorem 5.3 in [2] that

$$ \bigl(\mathcal{D}^{\alpha _{0}}_{a + , \mu }f \bigr) (x) = \frac{x^{- \mu }}{\Gamma (1 - \alpha _{0})} \biggl[f_{0}(a) \biggl( \log \frac{x}{a} \biggr)^{- \alpha _{0}} + \int _{a}^{x} \biggl(\log \frac{x}{t} \biggr)^{- \alpha _{0}} f_{0}'(t) \,d t \biggr], $$

where $f_{0}(x) = x^{\mu }f(x) \in \operatorname{AC}[a, b]$. We first claim that $(\mathcal{D}^{\alpha _{0}}_{a + , \mu }f)(x) \in X_{\mu }(a, b)$. Indeed,

$$ \int _{a}^{b} x^{\mu - 1} x^{-\mu } \biggl(\log \frac{x}{a} \biggr)^{- \alpha _{0}} \,d x = \int _{a}^{b} \biggl(\log \frac{x}{a} \biggr)^{- \alpha _{0}} \,d \biggl(\log \frac{x}{a} \biggr) = \frac{ (\log \frac{b}{a} )^{1 - \alpha _{0}}}{1 - \alpha _{0}} < \infty . $$

Similarly,

$$ \frac{x^{-1}}{\Gamma (1 - \alpha _{0})} \int _{a}^{x} \biggl(\log \frac{x}{t} \biggr)^{- \alpha _{0}} f_{0}'(t) \,d t \in X_{\mu }(a, b) $$

by noting that $f_{0}'(t) \in L[a, b]$ and

$$\begin{aligned}& \frac{1}{\Gamma (1 - \alpha _{0})} \int _{a}^{b} \frac{1}{x} \biggl\vert \int _{a}^{x} \biggl(\log \frac{x}{t} \biggr)^{- \alpha _{0}} f_{0}'(t) \,d t \biggr\vert \,d x \\& \quad \leq \frac{1}{\Gamma (1 - \alpha _{0})} \int _{a}^{b} \bigl\vert f_{0}'(t) \bigr\vert \,dt \int _{t}^{b} \biggl(\log \frac{x}{t} \biggr)^{- \alpha _{0}} \,d \biggl(\log \frac{x}{t} \biggr) = K \int _{a}^{b} \bigl\vert f_{0}'(t) \bigr\vert \,dt, \end{aligned}$$

where

$$ K = \frac{1}{\Gamma (2 - \alpha _{0})} \biggl(\log \frac{b}{a} \biggr)^{1 - \alpha _{0}}. $$

For $u \in X_{\mu }(a, b)$, equation (3) turns out to be

$$ a_{n} \bigl(\mathcal{J}^{\alpha _{n} - \alpha _{0}}_{ a + , \mu } u \bigr) (x) + \cdots + a_{1} \bigl(\mathcal{J}^{\alpha _{1} - \alpha _{0}}_{ a + , \mu } u \bigr) (x) + u(x) = \bigl(\mathcal{D}^{\alpha _{0}}_{a + , \mu }f \bigr) (x) $$

by applying the fractional differential operator $\mathcal{D}^{\alpha _{0}}_{a + , \mu }$ to both sides. Then by Theorem 2.1 we have

8 $$\begin{aligned} u(x) = & \sum_{k = 0}^{\infty }(-1)^{k} \sum_{k_{1} + \cdots + k_{n} = k} \binom{k}{k_{1}, k_{2},\ldots, k_{n}} a_{n}^{k_{n}} \cdots a_{1}^{k_{1}} \\ & {}\cdot \bigl(\mathcal{J}^{k_{n} (\alpha _{n} - \alpha _{0}) + \cdots + k_{1} ( \alpha _{1} - \alpha _{0}) }_{ a + , \mu } \mathcal{D}^{\alpha _{0}}_{a + , \mu }f \bigr) (x). \end{aligned}$$

To remove the differential operator $\mathcal{D}^{\alpha _{0}}_{a + , \mu }$, we compute the Hadamard-type fractional integral of order $\alpha >0$ for the first term in $(\mathcal{D}^{\alpha _{0}}_{a + , \mu }f)(x)$:

$$\begin{aligned}& \mathcal{J}^{\alpha }_{ a + , \mu } \frac{f_{0}(a) t^{- \mu }}{\Gamma (1 - \alpha _{0})} \biggl(\log \frac{t}{a} \biggr)^{- \alpha _{0}} \\& \quad = \frac{f_{0}(a)}{\Gamma (1 - \alpha _{0}) \Gamma (\alpha )} \int _{a}^{x} \biggl(\frac{t}{x} \biggr)^{\mu } \biggl(\log \frac{x}{t} \biggr)^{ \alpha - 1} t^{-\mu } \biggl(\log \frac{t}{a} \biggr)^{- \alpha _{0}} \frac{d t }{t} \\& \quad = \frac{f_{0}(a) x^{-\mu }}{\Gamma (1 - \alpha _{0}) \Gamma (\alpha )} \int _{a}^{x} \biggl(\log \frac{x}{t} \biggr)^{ \alpha - 1} \biggl( \log \frac{t}{a} \biggr)^{- \alpha _{0}} \frac{d t }{t}. \end{aligned}$$

Making the change of variable

$$ \tau = \frac{\log (t/a) }{\log (x/a)}, $$

we get

$$\begin{aligned} \int _{a}^{x} \biggl(\log \frac{x}{t} \biggr)^{ \alpha - 1} \biggl( \log \frac{t}{a} \biggr)^{- \alpha _{0}} \frac{d t }{t} = & \biggl( \log \frac{x}{a} \biggr)^{\alpha - \alpha _{0}} \int _{0}^{1} (1 - \tau )^{\alpha - 1} \tau ^{- \alpha _{0}} \,d \tau \\ =& \biggl(\log \frac{x}{a} \biggr)^{\alpha - \alpha _{0}} B(\alpha , 1 - \alpha _{0})\\ =& \biggl(\log \frac{x}{a} \biggr)^{\alpha - \alpha _{0}} \frac{\Gamma (\alpha ) \Gamma (1 - \alpha _{0})}{\Gamma (\alpha + 1 - \alpha _{0})}, \end{aligned}$$

where *B* denotes the beta function. Hence

9 $$ \mathcal{J}^{\alpha }_{ a + , \mu } \frac{f_{0}(a) t^{- \mu }}{\Gamma (1 - \alpha _{0})} \biggl(\log \frac{t}{a} \biggr)^{- \alpha _{0}} = \frac{f_{0}(a) x^{- \mu }}{\Gamma (\alpha + 1 - \alpha _{0}) } \biggl( \log \frac{x}{a} \biggr)^{\alpha - \alpha _{0}}. $$

The second term in $(\mathcal{D}^{\alpha _{0}}_{a + , \mu }f)(x)$ is

10 $$\begin{aligned}& \frac{1}{\Gamma (1 - \alpha _{0})} \int _{a}^{x} x^{- \mu } \biggl( \log \frac{x}{t} \biggr)^{- \alpha _{0}} f_{0}'(t) \,d t \\& \quad = \frac{1}{\Gamma (1 - \alpha _{0})} \int _{a}^{x} \biggl( \frac{t}{x} \biggr)^{\mu } \biggl(\log \frac{x}{t} \biggr)^{1 - \alpha _{0} - 1} \bigl[t^{- \mu + 1} f_{0}'(t) \bigr] \frac{d t }{t} \\& \quad = \mathcal{J}^{1 - \alpha _{0}}_{ a + , \mu } \bigl(t^{- \mu + 1} f_{0}'(t) \bigr) = \mu \bigl(\mathcal{J}^{1 - \alpha _{0}}_{ a + , \mu } f \bigr) (x) + \mathcal{J}^{1 - \alpha _{0}}_{ a + , \mu } \bigl(t f'(t) \bigr) (x). \end{aligned}$$

Therefore the solution immediately follows by substituting equations (9) and (10) into equation (8). This completes the proof of Theorem 2.4. □

### Remark 2

It seems impossible to deal with the case $\alpha _{0} \geq 1$ along the same lines as $\mathcal{D}^{\alpha _{0}}_{a + , \mu }f \notin X_{\mu }(a, b)$ for $f \in \operatorname{AC}[a, b]$. Furthermore, $\mathcal{D}^{\alpha _{0}}_{a + , \mu }$ is not a bounded operator on $\operatorname{AC}[a, b]$. The single-term Hadamard-type integral equation

$$ \mathcal{J}^{\alpha }_{a+, \mu } u = f, \quad \alpha > 0, $$

was studied in [2] with the necessary and sufficient conditions given in Theorem 3.1.

## Conclusions

Using Babenko’s approach and Banach’s contraction principle, we have derived the uniqueness of solution for several Hadamard-type integral equations and related coupled system. The results obtained are new in the present configuration of integral equations.

## Data Availability

Not applicable.
